# Account of the Killing of Supt. Reeves

**Published:** 1892-01

**Authors:** 


### THE KILLING OF DR. REEVES.

At 12:30 p. m., on the 29th day of December, 1891, this community and the whole State of Texas were shocked by the occurrence of a tragedy at the State Lunatic Asylum, near Austin, of a most startling and deplorable character. Superintendent W. W. Reeves was shot down with a double-barreled gun in the hands of Henry Purnell, a citizen of Austin and a very recent inmate of the asylum.

Purnell is a son of Judge Purnell, a well-known and prominent citizen of Austin. He is about 38 years of age, and up to the time of his becoming deranged in his mind, about eight years ago, was a popular young man, well known and received in the best society. The following record is taken from the books at the asylum, the entry having been made when he was first received as a patient:

"Admitted, December, 1885; private patient; age, 31; American, and unmarried; occupation, a clerk; education, good; date of first attack, four years previous; number of attacks, two; duration of last attack, one month; habits and natural disposition, temperate, and somewhat eccentric; no insanity in his family history; physical health, feeble, form [diagnosis] melancholy acute. He rapidly gained his physical health, and was discharged March 3, 1886, restored. No evidence given of any delusions. He was considered neither homicidal nor suicidal.

"He was re-admitted July3, 1886, in feeble health, and remained until May, 1887, and was again discharged, restored.

"He was again re-admitted, October 22, 1888, as a public patient, and after being kept a few months was again discharged, restored.

"He was re-admitted in this institution March 31, 1891. He was discharged the last time November 1, 1891, against his wishes."

It appears from the record that he was deranged in his mind only at times, and during his lucid intervals he was permitted many liberties, being thought harmless; also that he was several times discharged as restored, and several times re-admitted upon the return of symptoms of his malady. The record does not show it, but there are those in Austin who know him well, who say that his mental derangement dates from the kick of a horse, on the head, about eight years ago; others say, from a spell of fever about that length of time ago. With a history of a kick of a horse on the head, it would seem that on first admission to the asylum an examination of the skull would have been made, and relief by surgical operation must surely have suggested itself. However,—the above is the record.

Dr. Reeves took charge in February, 1891. Purnell was a favorite with him, and he allowed him many privileges. In November last, he was discharged as restored, and, to all appearances, he was perfectly sane. Along about Christmas, however, Mr. John Thornton, of the Galveston *News*, met him, and enquired how he was getting along. Purnell replied: "I am not doing well, I ought to be in the asylum. I am afraid I will hurt somebody."

It does seem that upon the occurrence of such evidence of a relapse, especially coupled, as it was, with acts clearly showing his insanity; for instance, his sending telegraphic messages C. O. D. to the dead Vanderbilt and the dead Astor about their property—his friends should have taken him in charge, to *save him from himself*. But the warning was unheeded.

He sought employment in Austin, and finding none, went to the asylum and told Dr. Reeves that he could find no work by which to support himself, and asked permission to earn his board and bed by doing jobs at the asylum. Dr. Reeves promptly assigned him to a place as keeper of the stores. But during his leisure hours, Purnell, who had been accustomed to frequent the office under former administrations, perhaps to wait on the officers, got to loafing around the office, or sitting there, reading. Dr. Reeves told him that it did not look well, especially when visitors came, and asked him to go to his room to read, when he had nothing to do, but suggested he could be doing something to earn his board. Purnell left reluctantly, but repeated the offense, whereupon, it is said, Dr. Reeves spoke sharply to him, and ordered him to go to work.

Nothing was said to indicate that Purnell meditated revenge. But on the morning of December 29th, he telephoned from the asylum to Dr. Ralph Steiner, in town, asking the loan of his gun. Dr. Steiner and he had been boys together and young men together in society, previous to his misfortune, and the doctor naturally felt a great sympathy for him, and believing him to be perfectly sane the greater part of the time, and harmless, readily consented. Purnell came in to town, got the gun and some shells, loaded it, and returned to the asylum. He entered the building at the main entrance, passing some photographers in the yard who were preparing to take a picture of the building. In reply to questions from them, he said he was going hunting, and passed on. Entering the main building, he went to the water cooler in the hall and got a glass of water, went to the kitchen and made a bag for birds, put his shells in it, and returned to the front door and out upon the stoop, at top of flight of some fifteen stone steps which lead from the ground to main entrance. Just as he reached the stoop Dr. Reeves arrived at the bottom of the steps, coming from his residence, which is in the front part of the asylum grounds. It is supposed he did not see Purnell. Purnell, standing at the head of the steps, without a word, raised the gun and fired one barrel point blank at the doctor, who was within about fifteen steps of him; the charge of buck shot entering the breast, caused death instantaneously. Dr. Reeves did not speak, but fell forward on the steps, dead.

Purnell passed the dead body and the photographers, on his way out. No one made any attempt to arrest him, and in reply to a question, "Henry, whom have you shot?" replied, "I had to kill Dr. Reeves." He got into the electric car just outside the asylum grounds, and came into the city, going directly to the court house, where he surrendered himself to the sheriff. He is now in jail. When questioned, he said he was "as sane as you are," and stated that he had good reasons for the killing of Dr. Reeves, which he did not pretend to deny.

TRAVIS COUNTY MEDICAL SOCIETY, at a regular meeting, in Austin, January 6th, unanimously adopted the following resolutions on the death of Dr. Reeves:

WHEREAS, Dr. W. W. Reeves, late Superintendent of the State Lunatic Asylum, a member of this Society, was foully assassinated on the 29th of December ult.,

*Resolved*, That in the death of Dr. Reeves, cut down in the prime and vigor of mature and happy manhood, on the threshold of a brilliant and promising career of active usefulness, Travis

County Medical Society has lost one of its brightest, most genial and accomplished fellows,—a man whom we were proud to number amongst our brotherhood and to call "friend"; that the medical profession at large, and the cause of science, have lost one of their brightest ornaments and most energetic and useful laborers, society a versatile and gifted member, a man above reproach in every relation of life. He was a high-minded and honorable gentleman, whose place in a profession which he adorned, and in society, where he shone conspicuous for grace, gallantry and high-born courtesy, will be forever vacant; but in the hearts and memory of his admiring and attached constituency his virtues and many excellencies will live forever. His family, to whom this Society tenders its most sincere condolence, has been bereft of a model husband and father.

*Resolved*, That a copy of these resolutions be sent to Mrs. Reeves, a copy be furnished the medical and secular press, and also spread upon the minutes of the Society.

*Resolved*, That the members of this Society wear the usual badge of mourning for thirty days, in respect to the memory of our departed brother.

